# Patient-Centered Communication: Incorporating Principles of Dialogic Practice and Family Centered Rounds on an Inpatient Psychotic Disorders Unit

**DOI:** 10.1007/s10597-024-01398-w

**Published:** 2024-12-07

**Authors:** Adrienne T. Gerken, Dost Öngür, Soo Jin Kim, Boyu Ren, Thomaskutty Idiculla, Joseph Stoklosa

**Affiliations:** 1 Department of Psychiatry and Human Behavior, Thomas Jefferson University Hospital, 33 South 9th St., Suite 210, Philadelphia, PA USA; 2 McLean Hospital, Belmont, MA USA; 3 Hartford Hospital, Hartford, CT United States of America

**Keywords:** Psychotic disorders, Patient-centered care, Schizophrenia, Patient care team, Mental health services

## Abstract

This study examined the impact of Patient-Centered Communication (PCC), Open Dialogue-inspired changes to rounding practices and culture, on patient perceptions of care on an inpatient psychotic disorders unit. A retrospective cohort analysis was conducted based on medical records, restraint and seclusion records, and hospital Perceptions of Care (PoC) surveys. The analysis compared data from 6-month periods before and after implementation of PCC to quantify whether the implementation of PCC was associated with more positive care ratings. There were trends toward improvement suggestive of an effect on patients’ perception that their care was adequately explained, that they felt involved in care, that they felt respected, and that they were supported during hospitalization, although improvements did not achieve statistical significance. Greater improvement was observed for teams that incorporated a greater number of interventions. Dialogic practice-inspired changes on an acute inpatient unit may improve patient perceptions of inclusion and respect in their care.

**Supplementary Information:**

The online version contains supplementary material available at 10.1007/s10597-024-01398-w.

## Introduction

Inpatient psychiatric care presents a therapeutic dilemma: how can providers and patients create a positive working relationship that promotes recovery when inpatient care is fraught with shortcomings, including perceived coercion and fractured therapeutic relationships (Bolsinger et al., 2019; Wyder et al., 2015)? How can we answer the Institute of Medicine’s charge to provide care that is “respectful of and responsive to individual patient preferences, needs, and values” (Institute of Medicine, 2001) when that care is provided behind a locked door?

In 2015, the McLean Hospital Schizophrenia and Bipolar Disorder Inpatient Program, which focuses on people experiencing psychosis, sought to create a more person-centered model of inpatient care. Recognizing that individuals experiencing psychosis may distrust providers and that early therapeutic relationships may impact later treatment outcomes (Beauford et al., 1997), the unit focused on interventions to promote transparency and collaboration by incorporating principles and structures borrowed from Open Dialogue and Family-Centered Rounds.

Seeking a more person-centered model of care for psychosis, leaders on our inpatient unit were drawn to Open Dialogue, an approach to psychiatric care that originated in Finland. It consists of an integrated system of care and a person-centered therapeutic approach. The Open Dialogue system of care requires integration across inpatient and outpatient settings to provide immediate assistance and continuity of care, and is thus challenging to implement in the US healthcare system.

Open Dialogue also utilizes *dialogic practice*, a form of therapeutic communication, in provider, patient, and family interactions. Key features of dialogic practice include the presence of two or more therapists, participation of the patient and supports in all meetings and decisions, an open-ended and transparent approach, inclusion of multiple viewpoints, and space for “reflections” (conversations between providers) within the meetings (Olson et al., 2014).

Finnish outcomes data for Open Dialogue have focused on first-episode psychosis and functional recovery. Early studies indicated a reduced need for antipsychotic medication, low incidence of residual psychosis, decreased need for ongoing psychiatric care, and high rates of full-time employment or education (Seikkula et al., 2006, 2011; Seikkula & Olson, 2003). Over an average of 19 years of follow-up, a Finnish Open Dialogue cohort experienced lower total durations of hospitalization, reduced use of antipsychotic medication, and less need for disability payments compared with controls (Bergstrom et al., 2018). Open Dialogue (or dialogic practice) has been implemented in several other countries. Published research remains limited. Studies in outpatient settings have demonstrated increased understanding of clients’ concerns (Buus et al., 2017), high client satisfaction (Buus et al., 2017; Florence et al., 2021; Gordon et al., 2016; Wusinich et al., 2020), and improved clinical outcomes (Gordon et al., 2016). However, some studies also noted logistical challenges (Buus et al., 2017; Razzaque & Wood, 2015; Tribe et al., 2019), discomfort with shifting roles (Buus et al., 2017), ambiguity or lack of purpose (Wusinich et al., 2020), and mixed feelings among staff (Buus et al., 2017; Wusinich et al., 2020). Trust and honesty between providers and clients, careful adaptation of the model to local conditions, and flexibility in provider attitudes were seen as vital to successful implementation (Buus et al., 2017).

Few studies have looked at implementation of Open Dialogue on inpatient units. One of the most comprehensive implementations of Open Dialogue in the United States has occurred at Grady Memorial Hospital in Atlanta, where an Open Dialogue pathway serves individuals experiencing psychosis (Cotes et al., 2023). The team follows patients across outpatient and inpatient levels of care with high levels of fidelity to the Open Dialogue model. The Grady program demonstrated acceptability for clients as well as trends toward clinical improvement.

Open Dialogue-inspired changes were also implemented for an inpatient unit in Australia, where study authors noted significant implementation challenges despite support from unit staff, including conflict between existing hierarchies and organizational structures, as well as economic pressures. Notably, the decision to introduce Open Dialogue was “top-down,” with unit staff learning of the change after the decision had been made, and most of the unit’s psychiatrists did not engage with the model (Dawson et al., 2021; Lennon et al., 2023).

In January 2015, the McLean Hospital Schizophrenia and Bipolar Disorder inpatient unit implemented changes to its culture and rounding practices to create a more person-centered approach to care (Rosen & Stoklosa, 2016). Prior to these changes, the unit used a more traditional model of psychiatric inpatient care with two sets of rounds: one in which patients were interviewed by the team, and a second in which the team met to make treatment and discharge decisions without the patient present. The unit has a culture of continuous self-improvement, and staff noted that the traditional rounding practice had the potential to foster mistrust, alienating the team from patients. Through in-service meetings, the multidisciplinary unit staff learned about Open Dialogue and discussed how to create a more person-centered inpatient experience. In-service meetings included information about Open Dialogue and Family Centered Rounds and discussion of how these ideas could be implemented on the unit. This process is discussed in further detail in Rosen and Stoklosa (2016).

Rather than attempting to strictly implement Open Dialogue, the unit focused on integrating principles of dialogic practice. Using principles of dialogic practice, as well as elements of Family Centered Rounds (FCR), which is the standard of care for pediatric intensive care units (Davidson et al., 2017) and inpatient pediatrics (Committee on Hospital Care and Institute for Patient- and Family-Centered Care, 2012), the unit restructured rounds to include patients in assessment and treatment planning. Three of four teams implemented a dialogic “reflecting team” as part of rounds, with a focus on openness, tolerance of uncertainty, transparency, and inclusion of the patient perspective. For all four teams, nursing and group reports were read aloud during rounds with the patient present, and the teams engaged in open discussions of their assessment and plan using person-centered language. Patients were invited to share their thoughts and observations during rounds (Rosen & Stoklosa, 2016).

Principles of FCR were incorporated for the treatment-planning portion of daily rounds. Aspects of FCR have significant overlap with those of dialogic practice, but there is a greater emphasis on direct discussion of treatment planning in person-centered, non-medicalized language. The insurance-based US healthcare system requires daily documentation of treatment plans. Given this requirement, the unit felt that FCR represented a method of treatment planning that was congruent with dialogic practice and more person-centered than traditional inpatient treatment-planning models.

Cultural and structural changes were implemented concurrently: earlier and more frequent family meetings; use of person-centered language when patients are present, in notes, and in clinician-only spaces; and implementation of patient-requested groups.

**Fig. 1:**
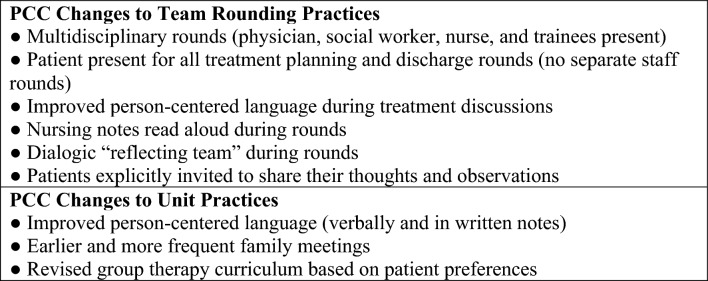
Elements of patient-centered communication (PCC)

The unit’s rounding and cultural changes were collectively termed Patient-Centered Communication (PCC). Elements of PCC are summarized in Fig. 1. The purpose of this study was to evaluate the effect of PCC changes on interpersonal aspects of patients’ perceptions of their care, with patient perceptions of being treated with respect and dignity as the primary outcome.

## Methods

### Study Setting

This was a retrospective cohort study based on data from the McLean Hospital Schizophrenia and Bipolar Disorder inpatient unit. During the study period, the unit was a 28-bed adult inpatient unit divided between four inpatient teams. The study population consisted of adult patients of all genders. Although the unit focuses on individuals with psychotic disorders, other diagnoses are represented on the unit and all patient records were included regardless of diagnosis.

The changes to the unit described above were implemented in January 2015. Outcome measures were compared between a 6 month period prior to these changes and a 6 month period after the changes, excluding a 3 month “transition period” centered around January 2015. To account for individual team differences, outcome measures were compared with each of the four teams serving as its own control. The study protocol was reviewed and approved by the Partners Healthcare (now Mass General Brigham) Institutional Review Board with a full waiver of informed consent.

### Measures

Data were obtained from the hospital’s electronic health records, restraint and seclusion records, and McLean Hospital Perceptions of Care (PoC) surveys.

#### Electronic Health Records

 Demographic information for all admissions during the two study periods, June 1 to November 30, 2014 (pre-PCC) and March 1 to August 31, 2015 (post-PCC) was extracted from the electronic health record. Information included the patient’s name, date of birth, medical record number, primary diagnosis at discharge, gender, age on admission, legal status on admission, dates of admission and discharge, attending of record, and inpatient team.

#### Restraint and Seclusion Data

 Hospital records of restraint and seclusion events from the two study periods were matched with the study subjects based on medical record number. The presence or absence of any restraint and seclusion event during the hospitalization was coded as “restraint,” as well as the number of such events if any occurred.

#### Perceptions of Care (PoC) Surveys

**Fig. 2:**
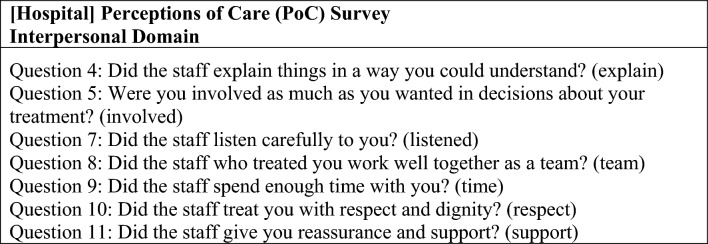
[Hospital] Perceptions of care survey: interpersonal domain questions

 The PoC survey is offered to all patients prior to discharge from McLean Hospital programs, including inpatient units. The survey consists of 18 items related to subjective experiences of care, including seven items that comprise the “interpersonal domain” of care (Fig. 2). Given the relevance of these items to the individual experience of care, including patient-staff interactions, the items on the interpersonal domain were selected as outcomes measures for this study. Question 10, which asks whether the patient feels they were treated with respect and dignity, was selected as the primary outcome prior to analysis. For each question in the interpersonal domain, a respondent could answer “Never (1),” “Sometimes (2),” “Usually (3),” or “Always (4).”

PoC data were extracted from the hospital PoC database and matched with subject hospitalization records for the two study periods (pre- and post-PCC).

### Statistical Analysis

This study was designed as a retrospective cohort analysis to compare outcomes before and after the PCC intervention, with each inpatient team serving as its own control. Subjects who completed the McLean Hospital PoC survey were included in the PoC analysis. Given that the unit’s model was novel and that covering attendings might not be familiar with this method of rounding, records were excluded if the attending of record was a covering attending. A small number of subjects completed two surveys for the same admission; in these cases, only the first survey for that admission was included. Missing values constituted < 0.1% of all data points for the interpersonal domain questions.

PoC data responses were skewed toward a response of “Always (4)” for both the pre- and post-PCC study periods. For this reason, and because the hospital’s goal is to consistently provide care that meets the “Always (4)” response threshold, PoC data outcomes were initially dichotomized to test the difference in the probability of answering “Always (4)” versus any other response.

Dichotomized PoC data were analyzed using logistic regression for a binary outcome, where the main effect of interest is the pre–post time effect. The analysis allowed for team effect, as well as time-team interaction effect. The analysis was completed twice to account for multiple admissions: once using only the subject’s first visit for the study period, then again to include all visits. To account for multiple comparisons, the p-value threshold for significance was assigned at 0.005, with a suggestion of effect for a p-value of 0.10 or less. Analyses were controlled for potentially confounding demographic variables including primary discharge diagnosis, presence or absence of a personality disorder diagnosis, gender, age on admission, legal status on admission, and length of stay (mean and median).

A follow-up analysis using ordered logistic regression was performed to examine whether the dichotomization of the PoC data leads to significantly different results and to quantify whether the implementation of PCC is associated with an increased likelihood to more positive ratings of care, not restricted to only the contrast between “Always” and others. The original PoC responses, which are treated as ordinal, were used as the outcome and the same set of outcomes as well as covariates were considered as in the dichotomized analyses. The same p-value thresholds (0.005 for significance and 0.1 for suggestion of effect) were used for the ordinal logistic regression.

In addition to our primary outcome, presence or absence of any restraint and length of stay were considered as secondary outcomes. *T *tests were used to compare the mean length of stay, comparing pre- and post-PCC periods and the mean number of restraints. Statistical analyses were performed with RStudio.

## Results

### Population Characteristics

During the study periods, a total of 715 patients were admitted to the AB2 inpatient unit, with similar numbers in the pre-PCC (*n* = 358) and post-PCC (*n* = 357) periods. PoC surveys were received for 409 admissions, reflecting a completion rate of 57%. Excluding covering attendings and duplicate records, 381 PoC surveys were available for analysis. Population characteristics for each team at time points 1 and 2 are presented in Table 1.

**Table 1:** Population characteristics of subjects

	Time 1 (Pre-PCC)						Time 2 (Post-PCC)					
	Team 1	Team 2	Team 3	Team 4	p-value	All	Team 1	Team 2	Team 3	Team 4	p-value	All
Number (n)	51	32	56	51	-	190	49	49	51	42	-	191
Age (years)	32.3	32.5	34.4	33.3	0.854	33.2	30.6	36.1	34.4	30.0	0.055	32.9
LOS (days)	13.5	13.1	14.9	13.2	0.921	13.8	13.7	15.4	13.5	11.2	0.517	13.5
Female (%)	35%	41%	45%	39%	0.690	40%	35%	39%	57%	43%	0.133	44%
Voluntary (%)	78%	91%	84%	86%	0.606	84%	79%	76%	78%	88%	0.580	80%

For continuous variables, a one-way ANOVA was used for the test and a Fisher’s exact test was used for the binary variables

P-values indicate whether the characteristics are significantly different across teams at a given time point.

### Perceptions of Care

After accounting for team effects, multiple visits, and demographic variables, the effect of the pre-post PCC indicator on the odds of choosing “Always (4)” were close to 2 for most teams on most questions (Table 2), indicating that respondents were approximately twice as likely to indicate “Always (4)” after implementation of PCC. There was no significant difference between the first analysis (initial visits only) and the second analysis (all visits, including readmissions).

**Table 2:** Effect of PCC from logistic regression allowing for main effect pre/post PCC and team and their interaction

Question	Odds ratio (effect of PCC)	*p* value	Suggestive modification by team
4 (explain)	2.08	0.08*	Yes; Team 1 improved vs. Team 3, *p* = 0.02
5 (involved)	2.13	0.07*	Yes; Team 1 improved vs. Team 3, *p* = 0.05
7 (listened)	1.50	0.33	No
8 (team)	1.71	0.19	No
9 (time)	1.15	0.72	No
10 (respect)	2.08	0.10*	No
11 (support)	2.41	0.04*	Yes; Team 1 improved vs. Team 3, *p* = 0.04

* *p* ≤ 0.10 (suggestive effect); ** *p* < 0.005 (significant effect)

OR reported for the reference team 1. Effect of PCC on likelihood of choosing “Always,” allowing for main effect (pre/post PCC), team, and their interaction. OR reported versus the reference (Team 1)

Table S1 reports the results from the second analysis. Similar trends, although at a finer resolution, were observed in results from the ordered logistic regression. That is, the odds of choosing a more positive rating of care, including that of choosing “Always” versus others, increased after the implementation of PCC (see Table S1). However, the p-values of some of the questions were higher than the corresponding p-values in the dichotomized analyses.

**Fig. 3:**
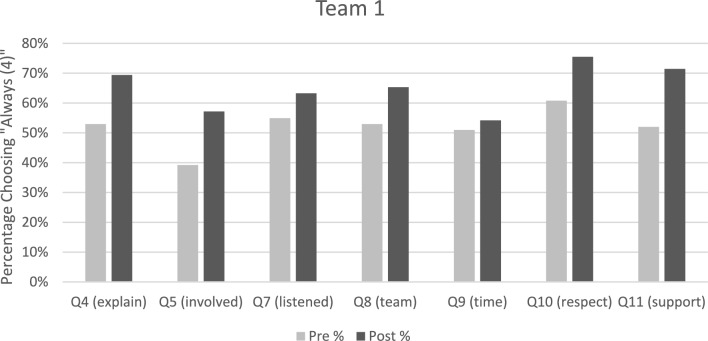
Comparison of pre- and post-PCC outcomes for Team 1, indicating the percentage of subjects choosing “always” for each item

Team effects emerged from the PoC data analysis. Three of the four teams (Teams 1, 2, and 4) showed a trend toward improvement between pre-PCC and post-PCC ratings. This tendency was strongest for Team 1, which had incorporated all PCC changes (Fig. 3). Teams 2 and 4 were statistically similar to Team 1. Team 3 incorporated the fewest PCC changes. Significant differences emerged between Team 1 and Team 3. Most of these effect modifications were also detected in the ordered logistic regression, with the only exception of Question 11, which asks about patient support (see Table S1).

### Additional Metrics

Mean length of stay for all teams was similar between the pre- and post-PCC periods. Although the absolute number of restraints decreased from 83 in the pre-PCC period to 46 in the post-PCC period, this change was driven by the presence of one individual patient in the pre-PCC period and the overall likelihood of any restraint did not differ significantly between the two periods.

## Discussion

To our knowledge, this is the first published report regarding outcomes of Open Dialogue-inspired changes on an inpatient psychiatric unit in the United States, and the first report worldwide on an inpatient unit specific to psychosis. Congruent with the goal of providing more person-centered care, the outcomes reported here are based on patient experiences of their care, including perceptions of respect and dignity.

While none of the improvements in PoC items reached statistical significance for multiple comparisons, there were trends toward improvement suggestive of an effect in several domains, including patients’ perception that their care was adequately explained to them and that they felt involved in their care. There were also suggestions of effect in terms of patients’ perceptions of respect and dignity, and in feeling supported during hospitalization.

Although the retrospective nature of this analysis precluded a fidelity measure, the team that included the most elements of dialogic practice saw the greatest improvements in PoC interpersonal domain scores, particularly when compared with the team that made the fewest changes. Time spent in rounds was not measured, though the perception of most staff members was that overall time spent in rounds decreased through the elimination of the separate treatment-planning rounds.

At a minimum, implementing these person-centered changes, including practices that might be considered radical in an acutely ill patient population—such as reading nursing notes aloud during rounds and sharing clinician assessments openly with patients—showed no evidence of worsening outcomes.

Notably, these positive results were achieved in the context of a unit that emphasizes an appropriate culture for person-centered interventions. As described by Buus and colleagues (2021), these characteristics include trust and honesty between staff, vulnerability, flexibility, and willingness to forego traditional hierarchical roles. The unit changes were collaboratively developed between interdisciplinary staff, rather than using a “top-down” approach. Concurrent changes, including staff training in person-centered language (critical before nursing notes can be read aloud), likely played a role in addition to the PCC model.

Strengths of the study include a large data set and the ability to compare the same teams before and after PCC implementation. Limitations of this study are related to its retrospective nature and include a lack of fidelity measures to account for between-team outcome differences. Future studies could include fidelity metrics for the PCC model, stratification of results by diagnosis, and additional outcomes measures that are specific to the PCC model. Further research could use an implementation science approach, starting with qualitative analysis of patient and provider perspectives. This research could include better identifying the cultural factors that allow adoption and predict fidelity to the model, with the goal of improving patient centered outcomes.

## Conclusions

Implementation of practices inspired by Open Dialogue on an acute inpatient unit are feasible and may improve patient experiences of inclusion and respect. These practices include the implementation of a dialogic reflecting team in rounds, transparency with respect to team notes and assessments, full integration of treatment and discharge planning into team rounds, and frequent family meetings. These changes are best implemented in the context of a unit culture of adaptability and flexibility, and that is responsive to the needs of patients and families.

Our results and experience support the idea that traditional models of inpatient care can be adapted to more person-centered practices that are more respectful, collaborative, and inclusive. We urge inpatient units to consider the implementation of person-centered practices, such as dialogic practice, in the context of unit- and patient-specific needs.

## Supplementary Information

Below is the link to the electronic supplementary material.

Supplementary file1 (DOCX 15 KB)
