# Supplementary material for: Patient-Centered Communication: Incorporating Principles of Dialogic Practice and Family Centered Rounds on an Inpatient Psychotic Disorders Unit
Source: Community Ment Health J. 2024 Dec 7;61(2):394–400. doi: 10.1007/s10597-024-01398-w (PMC11772458; doi:10.1007/s10597-024-01398-w)
Supplement: Supplementary file 1 — Supplementary file1 (DOCX 15 KB) [file 10597_2024_1398_MOESM1_ESM.docx]

**TABLE S1.** Effect of PCC on likelihood of choosing higher level of endorsement, allowing for main effect (pre/post PCC), team, and their interaction. OR reported indicates the comparison between any pair of consecutive response level and with respect to the reference group (Team 1)

| **Question** | **Odds Ratio**  **(Effect of PCC)** | **p value** | **Suggestive modification by team** |
| --- | --- | --- | --- |
| **4 (explain)** | 1.92 | 0.116 | Yes; Team 1 improved vs. Team 3, p = 0.006 |
| **5 (involved)** | 2.10 | 0.056* | Yes; Team 1 improved vs. Team 3, p = 0.015 |
| **7 (listened)** | 1.41 | 0.40 | No |
| **8 (team)** | 1.43 | 0.38 | No |
| **9 (time)** | 1.16 | 0.71 | No |
| **10 (respect)** | 1.83 | 0.17 | No |
| **11 (support)** | 2.05 | 0.08* | No |

* p ≤ 0.10 (suggestive effect); ** p < 0.005 (significant effect)
